# Metabolic Spectrum of Liver Failure in Type 2 Diabetes and Obesity: From NAFLD to NASH to HCC

**DOI:** 10.3390/ijms22094495

**Published:** 2021-04-26

**Authors:** Hyunmi Kim, Da Som Lee, Tae Hyeon An, Hyun-Ju Park, Won Kon Kim, Kwang-Hee Bae, Kyoung-Jin Oh

**Affiliations:** 1 Metabolic Regulation Research Center, Korea Research Institute of Bioscience and Biotechnology (KRIBB), 125 Gwahak-ro, Yuseong-gu, Daejeon 34141, Korea; khm7607@kribb.re.kr (H.K.); dasom89@kribb.re.kr (D.S.L.); anth0291@kribb.re.kr (T.H.A.); h18509@kribb.re.kr (H.-J.P.); wkkim@kribb.re.kr (W.K.K.); 2 Department of Functional Genomics, KRIBB School of Bioscience, Korea University of Science and Technology (UST), 217 Gajeong-ro, Yuseong-gu, Daejeon 34141, Korea

**Keywords:** non-alcholic fatty liver disease (NAFLD), non-alcoholic steatohepatitis (NASH), hepatocellular carcinoma (HCC), type 2 diabetes, obesity, adipokines, hepatokines

## Abstract

Liver disease is the spectrum of liver damage ranging from simple steatosis called as nonalcoholic fatty liver disease (NAFLD) to hepatocellular carcinoma (HCC). Clinically, NAFLD and type 2 diabetes coexist. Type 2 diabetes contributes to biological processes driving the severity of NAFLD, the primary cause for development of chronic liver diseases. In the last 20 years, the rate of non-viral NAFLD/NASH-derived HCC has been increasing rapidly. As there are currently no suitable drugs for treatment of NAFLD and NASH, a class of thiazolidinediones (TZDs) drugs for the treatment of type 2 diabetes is sometimes used to improve liver failure despite the risk of side effects. Therefore, diagnosis, prevention, and treatment of the development and progression of NAFLD and NASH are important issues. In this review, we will discuss the pathogenesis of NAFLD/NASH and NAFLD/NASH-derived HCC and the current promising pharmacological therapies of NAFLD/NASH. Further, we will provide insights into “adipose-derived adipokines” and “liver-derived hepatokines” as diagnostic and therapeutic targets from NAFLD to HCC.

## 1. Introduction

Type 2 diabetes is the main public health problem in terms of global epidemic and pandemic diseases [1,2]. It is closely related with the worldwide epidemic of obesity, and approximately 75% of type 2 diabetes is related with obesity [3,4]. The relationship between type 2 diabetes and obesity is further explained by the descriptive term of “Diabesity” [5]. Actually, the modern sedentary lifestyle contributes to weight gain by promoting excessive food intake and even adding physical inactivity [6,7]. For that reason, chronic metabolic diseases such as type 2 diabetes and obesity have been increasing globally.

**Figure 1:**
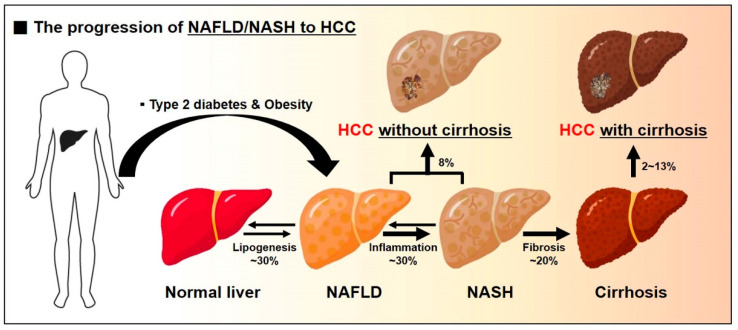
Type 2 diabetes and obesity aggravate the progression of NAFLD/NASH to HCC. Clinically, type 2 diabetes coexists with NAFLD, and it aggravates NAFLD to more severe forms of NASH, hepatocirrhosis, and HCC, leading to a metabolically worse phenotype.

Together with obesity and type 2 diabetes, non-alcoholic fatty liver (NAFLD) is the most common liver disease, and is observed in approximately 30% of the general population [8,9,10]. NAFLD is characterized by hepatic triglyceride (TG) accumulation and insulin resistance [11,12]. It is the hepatic manifestation of metabolic syndrome and is a spectrum of conditions ranging from benign hepatic steatosis to non-alcoholic steatohepatitis (NASH) [13]. That is, it is broadly categorized into non-alcoholic fatty liver (NAFL) and NASH [14]. NAFL is marked by isolated steatosis, and NASH is characterized by steatosis, lobular inflammation (inflammatory cell infiltration), and hepatocellular ballooning in the presence or absence of fibrosis [15]. NASH, the more aggressive form of NAFLD, could develop into progressive fibrosis, and is directly associated with the risk of developing hepatocellular carcinoma (HCC), which could be a major cause of morbidity and mortality induced from liver failure (Figure 1) [8]. The prevalence of NASH is approximately ~30% for patients with NAFLD [16,17,18,19]. Approximately 20% of NASH patients with fibrosis progress to cirrhosis [20]. Liver cirrhosis is present in only 50% of patients with NAFLD-related HCC [21,22]. The incidence of NAFLD-related HCC without cirrhosis is approximately 8% of all HCC cases [23,24]. The incidence rate for HCC in NAFLD/NASH with cirrhosis ranges from 2% to 13% (Figure 1) [25,26].

Clinically, NAFLD coexists with type 2 diabetes and obesity, and it exerts a synergistic effect, leading to more severe liver failures [27]. The prevalence rate of NAFLD is estimated to be approximately 75% in patients with type 2 diabetes and about 90% in the obese population, which show the strong relationship of NAFLD with type 2 diabetes and obesity [28,29,30,31]. NAFLD plays an important role in increases of the incidence of type 2 diabetes and its complications [28]. Type 2 diabetes also exacerbates NAFLD to more severe forms of NASH, fibrosis, and HCC (Figure 1) [30,32,33].

HCC is one of the most aggressive growing cancers [34,35]. Previously, hepatitis C virus (HCV) was thought to be the leading cause of HCC [36,37,38], but recent reports that showed up to 50% of newly diagnosed HCC patients are non-viral HCC [39,40], Therefore, NAFLD/NASH-derived HCC has been highlighted. The etiology of NAFLD/NASH-derived HCC is very complex and is related with various mechanisms such as cellular plasticity, inflammation, apoptosis, cell cycle, and cell death [41,42]. It is not easy to treat and improve HCC. Therefore, NAFLD/NASH treatment is required for prevention of irreversible chronic liver diseases such as cirrhosis and HCC. Unfortunately, there are no FDA-approved drugs and treatment methods yet.

In this review, we will discuss the pathogenesis of NAFLD/NASH and NAFLD/ NASH-derived HCC and the current promising pharmacological therapies of NAFLD/ NASH. Further, the initiation and progression of NAFLD can be affected by organokines secreted from metabolic organs under metabolic disturbance such as type 2 diabetes and obesity [43,44,45]. Therefore, we will focus on organokines that are secreted by the adipose tissues and liver, which are critical organs for the regulation of lipid metabolism. We will provide new insights into “adipokines” and “hepatokines” that can be potential diagnostic and therapeutic targets in NAFLD/NASH and NAFLD/NASH-derived HCC. They are thought to be able to be biological markers that can predict NAFLD severity from NAFLD to HCC.

## 2. Non-Alcoholic Fatty Liver Disease (NAFLD) and Non-Alcoholic Steatohepatitis (NASH)

### 2.1. Pathogenesis of NAFLD and NASH

#### 2.1.1. An Imbalance in Fatty Acid (FA) Metabolism

**Figure 2:**
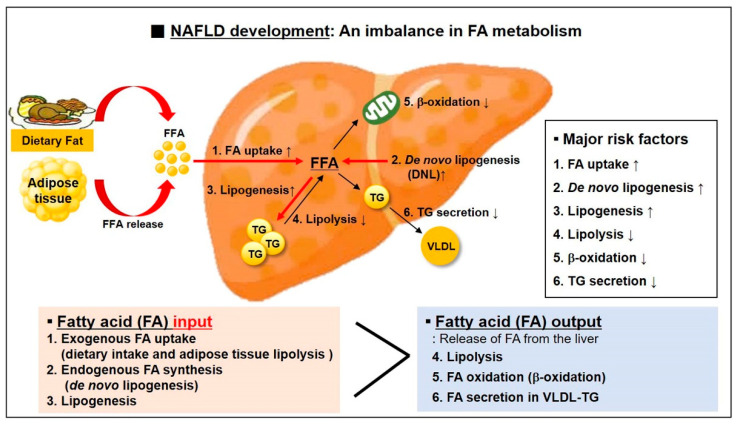
NAFLD development is caused by an imbalance in the intrahepatocellular fatty acid (FA) metabolism. Hepatic TG accumulation is promoted when the FA input is greater than the FA output in the liver. The greater part of FA taken up by liver is mainly derived from the lipolysis of subcutaneous adipose tissue TG. Another major source of FA in the liver is derived from de novo lipogenesis that converts excess glucose into FAs. On the other hand, the consumption of FA is possible through the signaling pathway involved in lipolysis, β-oxidation, and TG secretion (→: signaling pathways related with TG accumulation by FA, →: signaling pathways related with the consumption of FA).

NAFLD is the most common etiology of chronic liver diseases. NAFLD results from excessive triglyceride (TG) accumulation in the liver [11,12]. Therefore, the balance between fatty acid (FA) input and FA output is critical [46,47]. That is, NAFLD develops when the amount of “exogenous FA uptake (dietary intake and adipose tissue lipolysis)” and “endogenous FA synthesis (DNL: de novo lipogenesis)” in the liver is greater than “the release of FAs (FA oxidation, lipolysis, and FA secretion in very low density lipoprotein (VLDL)-TG)” from the liver (Figure 2).

The release of FAs from adipose tissue and the efficiency of FA uptake by liver are increased in approximately 59% of patients with NAFLD [48]. Hepatic FA uptake depends on plasma FA concentration and on hepatocellular capacity for FA uptake that is determined by the number and activity of specialized FA transporter and carrier proteins such as FA translocase (FAT/CD36), FA transport polypeptide (FATP), and FA binding protein (FABP) [49,50]. For example, hepatic expression of fatty acid translocase CD36 is markedly increased in subjects with NAFLD, and hepatic expression of FABP-4 and FABP-5 is closely associated with intrahepatic TG accumulation.

In approximately 26% of patients with NAFLD, the way to provides FA pool in liver is de novo lipogenesis (DNL) [51]. DNL is the metabolic process that synthesizes new FAs from excess glucose [52,53]. It is an important contributor toward hepatic lipid accumulation in the pathogenesis of NAFLD [52,53]. The effects result from activation of two transcription factors, sterol regulatory element binding protein-1c (SREBP-1c) and carbohydrate responsive element binding protein (ChREBP), boosted by insulin and glucose responses to dietary carbohydrates [54,55]. They play a synergistically important role in the coordinated regulation of hepatic DNL. In the remaining 15% of patients with NAFLD, FA pool is derived from dietary TG, which is associated with chylomicrons [48].

The most acceptable theory in the pathogenesis of NAFLD is the “two-hit” hypothesis [56]. The first hit is “insulin resistance” caused by excessive FA flux into the liver. The second hit is “inflammation”, associated with gut-derived endotoxin, oxidative stress, and mitochondria dysfunction. It is closely related with NAFLD progression toward NASH.

#### 2.1.2. Endotoxin Behavior

NALFD and other insulin resistance-related diseases are associated with activation of innate immune system, leading to chronic inflammation [57]. Recently, gut-derived endotoxins, such as lipopolysaccharide (LPS), have been proposed to have a critical role in liver inflammation as well as progression of chronic liver diseases [58]. Under normal conditions, endotoxin can be absorbed from the intestinal lumen into the portal vein system, and the absorbed endotoxin will be rapidly removed in the hepatic reticuloendothelial system, particularly Kupffer cells [59,60]. However, obesity, type 2 diabetes, and other nutrition and environmental factors can alter intestinal permeability for bacterial overgrowth and the resulting leaky mucosal barrier allows bacterial translocation, implicating the release of gut-derived endotoxin into the systemic circulation [61,62]. The invasive pathogens and harmful byproducts influence hepatic lipid accumulation and exacerbate pro-inflammatory and fibrotic processes [60].

Recently, the role of LPS from gut microbiota in the development of NAFLD and NASH has been attracting attention [63,64]. Circulating LPS levels, small intestinal permeability, and bacterial overgrowth are increased in patients with NALFD, and these factors are associated with the severity of hepatic steatosis [63,65,66]. Livers that directly receives blood from the portal vein are the main target of LPS, also known as endotoxin, and LPS-TLR4 is one of the critical pathways for NAFLD development. In mouse models, LPS infusion triggers hepatic steatosis and hepatic insulin resistance, as well as hepatic weight gain [67]. LPS exacerbates liver injury in mice fed a methionine-choline-deficient diet [68]. The LPS-binding protein (LBP)-CD14 complex activates Toll-like recpeotr4 (TLR4), which is an essential inflammatory cascade in the progression of NAFLD [69,70]. Loss of LBP attenuates inflammation-mediated liver damage [71]. TLR4 can activate NF-kB and release pro-inflammatory cytokines such as interleukin-1β (IL-1β), tumor necrosis factor-α (TNF-α), and IL-6 [72]. It can also recognize damage-associated molecular patterns (DAMPs) that are released from damaged cells, and mediates FA-induced inflammation [57,73]. As pharmacological therapies in NAFLD and NASH targeting the microbiome, there are IMM-24e (anti-LPS antibody), solithromycin (next-generation macrolide antbiotic), and TLR4 antagonist [74].

#### 2.1.3. Oxidative Stress

Chronic oxidative stress is one of the key mechanisms leading to liver injury in NAFLD. Oxidative stress is a general event that occur in NAFLD and NASH as result of excessive production of reactive oxygen species (ROS) [75,76]. ROS and lipid peroxidation can explain most of histological features of NAFLD and NASH [77,78]. In patients with hepatic steatosis, mitochondrial ROS oxidizes hepatic fat deposits, and ROS-induced expression of Fas-ligand can induce apoptosis [77,78]. Peroxidation of and intracellular membranes can directly trigger necrosis and apoptosis [77,78]. The degree of lipid peroxidation is correlated with the severity of steatosis and can explain the association between steatosis severity and the risk of necroinflammation and fibrosis in NASH [79,80,81]. ROS, which plays a key player in the pathogenesis of NASH, can lead to a self-perpetuating cycle of lipid peroxidation and can further generate ROS [82]. Lipid peroxidation products alter mitochondrial DNA and activate transcription factor nuclear facto-kB (NF-kB) that upregulates TNFα [83,84]. Resultingly, it further contributes to impaired mitochondrial respiration and increased ROS formation [83,84].

Increased mitochondrial β-oxidation of FFA is an important source of ROS in NAFLD and NASH [85]. Increased FFA flux in hepatic cells during early stages of NAFLD stimulate mitochondrial fatty acid oxidation (FAO), and it reflects an early effort of the liver compensatory mechanisms to inhibit liver fat accumulation and maintain lipid homeostasis [12]. In NAFLD and NASH, mitochondrial FAO is also increased or at least preserved as a compensatory response. The imbalance between mitochondrial FAO and electron transport chain (ETC) will contribute to ROS overproduction by increased electron leakage from the ETC [12,85,86]. ROS-induced lipid peroxidation leads to inflammation and hepatic fibrogenesis through the activation of hepatic stellate cells (HSCs) [87,88].

Recently, reliable circulating markers that can reflect oxidative stress in patients with NAFLD have been reported. Urinary 8-iso-prostaglandin F2α (8-iso-PGF2α) is known as a reliable indicator of oxidative stress in vivo [89,90], and soluble NOX2-derived peptide (sNOX2-dp) are also an acceptable marker, which is associated with ROS generation by activation of NOX2, a member of the NADPH oxidase family [91,92]. Elevated levels of urinary 8-iso-PGF2α and serum soluble NOX2-derived peptide are considered as a reliable indicator of oxidative stress in chronic inflammation and metabolic diseases [93,94,95]. They also can be used as markers of oxidative stress for prediction of the severity of liver damage in NAFLD [96,97]. LPS is an important outer membrane component of gram-negative bacteria that induces accelerated inflammation and oxidative stress [98,99]. Elevated levels of circulating NOX2 and LPS in NAFLD patients suggest the potential role of gut-derived LPS in systemic NOX2 activation [100]. Further, sNOX2-dp levels are positively related with the histological grading of steatosis, inflammation, ballooning, fibrosis, and NAFLD activity score [100]. Gut-derived LPS can stimulate TLR4, and TLR4-mediated NOXs activation can generate ROS by macrophage infiltration [101]. It can contribute to hepatic steatosis and insulin resistance [101].

**Figure 3:**
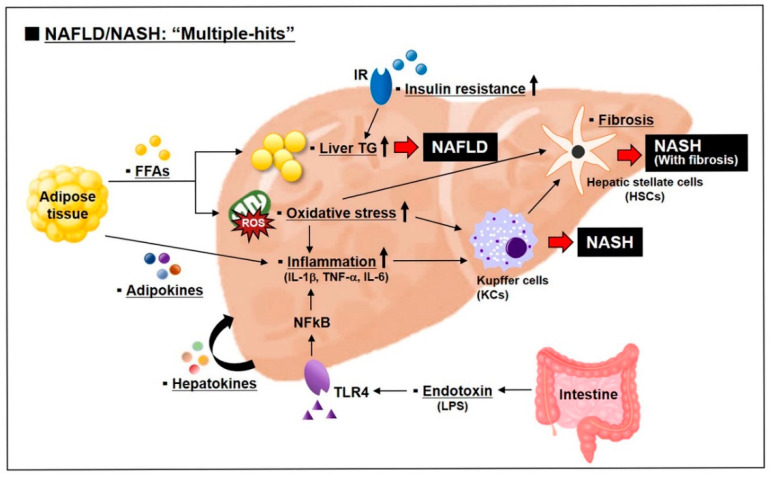
Multiple-hits pathogenesis of NAFLD and NASH. NAFLD begins with hepatic lipid accumulation and insulin resistance, and progresses to NASH with the concert of various factors such as inflammation, endotoxin, organokines (adipokines and hepatokines), and oxidative stress. (▪: Factors related with multiple-hits).

However, the variety of metabolic changes occurred in NAFLD are insufficient to be explained only by the “two-hit” hypothesis. Most metabolic disorders such as obesity, type 2 diabetes, metabolic syndrome, and dyslipidemia are the risk factors for NAFLD development. Recently, it has been thought that the development and progression of NAFLD are induced by the “multiple-hits” involving various factors (Figure 3) [102,103]. The “multiple-hits” include bioactive molecules secreted from the adipose tissue, nutritional factors, and environmental factors [102].

### 2.2. Promising Therapies in NAFLD and NASH

**Figure 4:**
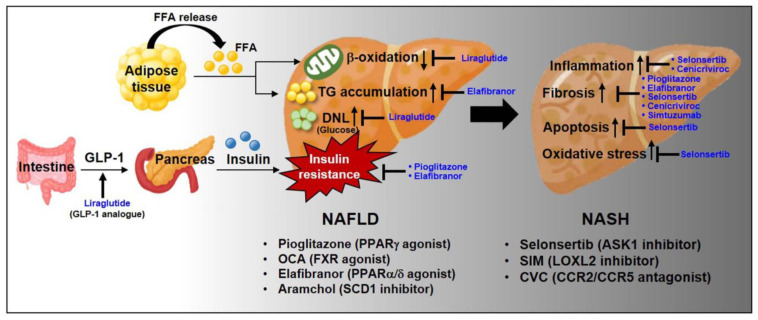
Current therapeutic targets for pharmacological treatment of NAFLD and NASH. There are no FDA-approved medications for patients with NAFLD/NASH so far. Currently, various pharmacological therapeutic candidates are being applied to the clinical trials. The illustration demonstrates the targeted pathway and phenotype for treatment of patients with NAFLD and NASH.

As recently recommended pharmacotherapies, it has been reported that pioglitazone and high dosage vitamin E effectively improve the histology of patients with NASH [104,105,106]. On the other hand, metformin does not recover liver histology of patients with NAFLD [107,108], and ursodeoxycholic acid (UDCA) does not improve liver histology, inflammation, or fibrosis of patients with NASH [109,110,111]. Below are some of pharmaco-therapeutic options that are in clinical trials or could be good candidates for NASH treatment (Figure 4). Additionally, the metabolic profile and liver histology-related efficacy of these promising drugs in humans are summarized in Table 1.

#### 2.2.1. Pioglitazone

Pioglitazone is one of the anti-diabetic agents of the thiazolidinediones (TZDs) class used in the management of type 2 diabetes [112]. TZDs is also known as “glitazones”. There are two TZDs, rosiglitazone and pioglitazone, which are currently approved by the FDA as monotherapy or combined therapies with metformin and sulfonylureas for managing type 2 diabetes [113]. TZDs, as insulin sensitizers, help regulate glycemia and insulin resistance [113]. The most important advantage of TZD is that it does not cause hypoglycemia with single therapy, and there are no contraindications for patients with renal disease [114].

TZDs regulates metabolic pathways by binding to the nuclear transcription factor peroxisome proliferator-activated receptor gamma (PPARγ) and modulating target gene expression [115]. The genes play a role in glucose metabolism, FA storage, and adipocyte differentiation [116]. In line with this, PPARγ agonists increase glucose transporter 4 (GLUT4, also known as SLC2A4) expression and translocation, inhibit TNF-α, and enhance insulin sensitivity in insulin-sensitive organs [117,118]. On the other hand, TZD therapy leads to weight gain as a side effect, because PPARγ receptors are highly expressed in adipocytes [119]. Increases in fat mass are exclusively limited to the subcutaneous adipose depot rather than the visceral spot [117,120]. They can be improved by treatment with metformin [121,122].

Recently, it was reported that the PPARγ agonist Pioglitazone has significant effects on NAFLD/NASH patients. In NASH patients, it improves liver fat accumulation and fibrosis [123,124]. In NASH patients with type 2 diabetes, it reduces hepatic steatosis, inflammation, and the serum alanine aminotransferase (ALT) and aspartate aminotransferase (AST) levels and improves the liver [125]. In rodent models, it reduces hepatic gluconeogenesis, and improves insulin sensitivity in the liver and other peripheral tissues [126]. It also improves hepatic fibrosis [126].

#### 2.2.2. Obeticholic acid (OCA, Also Known as INT-747; FXR Agonist)

OCA is a potent and selective agonist of farnesoid X receptor (FXR), a nuclear receptor that can regulate hepatic glucose and lipid metabolism, inflammation, and lipoprotein composition as well as bile acid synthesis [127,128]. In rodent models, OCA exerts the HSCs and macrophages anti-inflammatory and anti-fibrotic effects [129,130]. The transcriptional repressor small or short heterodimer partner (SHP) interacts with liver receptor homolog-1 (LRH-1), a positive regulator of CYP7A1 that encodes the rate-limiting enzyme in the classic bile acid synthesis pathways, and suppresses its transcriptional activity [131]. Exposure of HSCs to FXR ligands increases the transcriptional repressor SHP expression and reduces factors associated with liver fibrosis [130]. It is thought that an FXR-SHP regulatory axis plays an important role in regulating liver fibrosis. OCA-induced FXR activity is 100-fold more potent than human natural FXR agonist, chenodeoxycholic acid (CDCA) [132]. OCA increases insulin sensitivity and reduces markers related with hepatic inflammation and fibrosis in patients with type 2 diabetes and NAFLD [133]. OCA leads to weight loss in patients with NASH, and weight loss caused by OCA is shown to exert additively beneficial effects on serum aminotransferase and liver histology [134]. Additionally, it significantly improves fibrosis in NASH patients [135]. It is one of the most promising drugs for treating NASH and is now in phase 3 clinical trials [136].

#### 2.2.3. Elafibranor (GFT505; PPARα/δ Agonist)

PPARs are ligand-activated transcription factors of nuclear hormone receptor superfamily [137]. They are expressed in the liver, adipose tissue, skeletal muscle, heart, and kidney, and regulate metabolic pathways including β-oxidation and gluconeogenesis [136]. There are three nuclear receptor isoforms: PPAR alpha (α), PPAR delta (δ), and PPAR gamma (γ). PPARα promotes β-oxidation, reduces TG levels, and increases high-density lipoprotein (HDL) cholesterol [138]. It also inhibits NF-kB-induced inflammatory genes [138]. PPARα agonist, in forms such as fibric acid derivatives (fibrates), is broadly used for treatment of hypertriglycemia, whereas it has no significant effects on patients with NAFLD [139]. This is considered to be because PPARα receptors are present in other organs as well as the liver. Similar to PPARα, PPARδ increases FA oxidation and additionally reduces activation of macrophages and Kupffer cells because it is present in macrophages [140]. GW501516 is a synthetic PPARδ-specific agonist [141,142]. GW501516 might be considered as a promising therapy in clinical trials because of its potent efficacy, but it has safety concerns [143].

Elafibranor, also known as GFT505, is a dual PPARα/δ agonist [144]. It improves inflammation, apoptosis, and necroptosis in the NASH mouse model [145]. It reduced histopathologically hepatic steatosis and inflammation, and reduced fibrosis severity in both NAFLD/NASH and fibrosis mouse models [146,147]. It tends to reduce body weight, but not liver weight in diet-induced NAFLD/NASH rodent models [148,149]. In obese patients, it improves hepatic and peripheral insulin sensitivity [150]. Further, it inhibits proinflammatory (IL-1β, TNF-α, and F4/80) and profibrotic (transforming growth factor-β (TGF-β), tissue inhibitor of metalloproteinase 2, collagen type I, alpha 1, and collagen type I, alpha 2) markers in obese patients [151]. In addition, it decreases liver dysfunction markers such as ALT and alkaline phosphatase (ALP) [146,151]. It did not cause weight gain [144,152]. It is currently being evaluated in a pivotal phase 3 clinical trials in NASH patients [136].

#### 2.2.4. Arachidyl Amido Cholanoic Acid (Aramchol)

Aramchol is the liver targeted, oral stearoyl-CoA desaturase 1 (SCD1) inhibitor [153]. It is a novel fatty acid bile acid conjugate (FABACs) [153]. In rodents, aramchol affects liver fat metabolism by reducing FA synthesis and increasing β-oxidation [154,155]. Furthermore, aramchol activates cholesterol efflux by stimulating the ATP-binding cassette transporter A1 (ABCA1) [156]. In addition, it reduces inflammation and fibrosis in methionine and choline deficient (MCD) fed mice [153]. Additionally, it improves steatohepatitis and fibrosis by decreasing SCD1 levels and by regulating the transsulfuration pathway, leading to a rise in glutathione (GSH) levels and the glutathione disulfide (GSSG)/GSH redox couple to properly balance the redox environment [153]. Weight loss by aramchol treatment is known to stabilize within 1 week [153].

In in a phase 2 trial of patients with NAFLD, aramchol reduces the liver fat content and improves liver histology [157]. There was no significant toxicity as determined by the circulating ALT, AST, and alkaline phosphatase levels [157]. Because it targets both the general characteristics of NASH (excessive liver fat contents, lipotoxicity, and oxidative stress) and fibrosis [153], aramchol is currently being developed for the treatment of NASH and fibrosis. It is known that there were no significant changes in the body weight of NASH patients. Phase 3 clinical trials in patients with NASH and fibrosis were initiated in 2019 and are ongoing.

#### 2.2.5. Liraglutide (GLP-1R Agonist)

Glucagon-like peptide-1 receptor (GLP-1R) agonists are well established as an effective medication showing promising anti-diabetic effects in both animal models and patients with type 2 diabetes [158,159,160]. GLP-1 is a incretin hormone that is secreted from L-cells in the distal ileum and colon [161]. It stimulates the pancreas, leading to insulin biosynthesis and insulin secretion, and reduces glucagon production [162,163]. Endogenous GLP-1 is degraded within a few minutes by the dipeptidyl peptidase-4 (DPP-4) enzyme, but liraglutide works for a long time, with a half-life of 13 h [164].

Exenatide, a synthetic exendin-4, was the first GLP-1R agonist approved by the FDA in 2005 for the treatment of type 2 diabetes, as monotherapy or as add-on treatment to metformin and/or sulfonylurea where control was inadequate [165].

Liraglutide is the second GLP-1R agonist to be licensed the FDA in 2010 for the treatment of type 2 diabetes. It is also received FDA approval in 2020 as a treatment for obesity patients, based on its lasting weight loss benefits [166,167]. It has cardiovascular safety in treatment for weight management [168]. Liraglutide-induced anorexia is also related with glutamatergic POMC neuron, leading to weight loss [169]. In patients with NAFLD and NASH, it decreases liver fat contents and improves histological resolution and serum liver enzyme levels without worsening fibrosis [170,171]. It is thought that the effect of liraglutide on weight loss and reduced cardiovascular risk is critical for treatment of NAFLD because the development of NAFLD is based on lipotoxicity and insulin resistance [171,172]. As studies associated with NAFLD and NASH in rodents showed, liraglutide protects pancreatic β-cells from apoptosis through AKT-mediated survival signaling [173]. It improves insulin sensitivity by activating AMP-activated protein kinase (AMPK) and reduces liver steatosis by modulating lipid transport, β-oxidation, DNL, and autophagy [174,175,176].

#### 2.2.6. Selonsertib (ASK1 Inhibitor)

Ballooned hepatocytes, implicating activation of the apoptotic pathway, are a hall marker of NASH and fibrosis progression [177,178]. Selonsertib is a first-in-class inhibitor of the apoptosis signal regulating kinase 1(ASK1) [179]. It inhibits the phosphorylation and activation of ASK1 by binding to the catalytic kinase domain of ASK1. Recently, it has proposed as therapeutic potential for fibrotic diseases. In mouse models, ASK1, a serine/threonine signaling kinase, causes phosphorylation of p38 mitogen-activated kinase and c-Jun N-terminal kinase (JNK), leading to activation of stress response pathways that aggravate hepatic inflammation, apoptosis, and fibrosis [180,181,182]. In mouse models of NASH, it significantly improves not only liver steatosis and fibrosis associated with NASH but also cholesterol, bile acid, and lipid metabolism [180]. In phase 2 clinical trials of patients with NASH and stage 2–3 fibrosis, it has been shown to prevent inflammation, fibrosis, excessive apoptosis, and progression to cirrhosis [183]. On the other hand, phase 3 clinical trials of patients with NASH and advanced fibrosis were found to improve liver histology, but did not affect fibrosis regression [184,185]

#### 2.2.7. Simtuzumab (SIM, G6624)

SIM is a monoclonal antibody targeting the lysyl oxidase-like 2 (LOXL2) enzyme that catalyzes the crosslinkage of collagen and elastin, leading to remodeling of the extracellular matrix [186,187]. SIM binds to LOXL2 and inhibits its enzymatic activity [188]. As a result, it inhibits synthesis of growth factors including connective tissue growth factor (CTGF/CCN2) and TGFβ1, and reduces liver fibrosis [189]. In a mouse model with advanced fibrosis, SIM has an additive effect in combination with ASK1 inhibitor [183]. However, in phase 2b clinical trials of subjects with advanced fibrosis induced by NASH, it was no effect on improving fibrosis and cirrhosis confirmed by hepatic collagen content [190].

#### 2.2.8. Cenicriviroc (CVC; Dual CCR2/CCR5 Antagonist)

Liver inflammation is closely associated with chemokines that regulate activities and migration of hepatocytes and immune cells [191]. The C-C chemokine receptors CCR2 and CCR5 with their respective ligands (CCL2 and CLL3-5) are associated with the pathogenesis of liver inflammation and fibrosis for the development of NAFLD and NASH [191,192,193]. CCR2 and its ligand CCL2 enhances hepatic steatosis, macrophage accumulation, inflammation, and fibrosis [191]. Activated HSCs, a contributor for fibrosis, secretes CCL5. CCL5 exerts profibrotic activity in hepatocytes via its receptors CCR5 and induces lipid accumulation and pro-inflammatory factors [192].

CVC is a novel and potent antagonist of CCR2 and CCR5 that is currently in clinical development for treatment of liver fibrosis in patients with NASH [194,195]. CVC reduces levels of inflammation markers including IL-1β and IL-6 and exerts anti-fibrotic activities [194,195]. It received Fast Track designation by the FDA in 2015 as a promising therapy for NASH and liver fibrosis. In the phase 2b study of subjects with NASH and stage 2–3 fibrosis, CVC has shown improvement in liver fibrosis without worsening NASH [196]. Currently, phase 3 clinical trials are ongoing to evaluate and confirm the efficacy and safety of CVC for the treatment of liver fibrosis in patients with NASH [197].

### 2.3. Diagnostic and Therapeutic Targets in NAFLD and NASH: Adipokines

**Figure 5:**
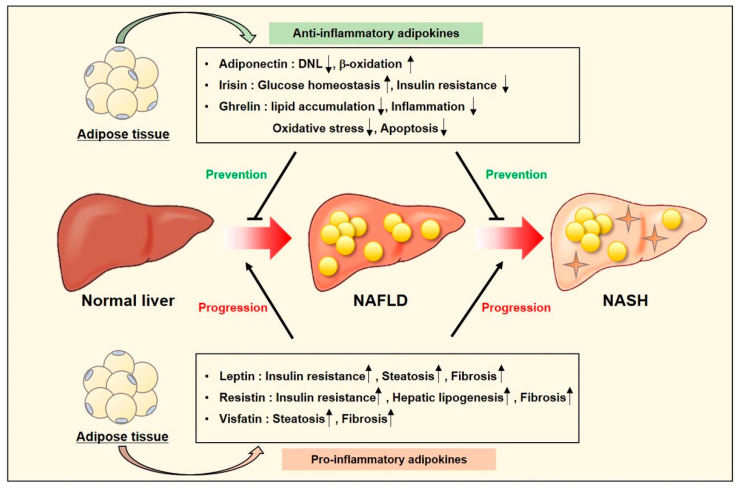
Adipokines as diagnostic markers and therapeutic targets in NAFLD and NASH. Adipokines that are secreted from adipose tissues are classified into anti-inflammatory adipokines and pro-inflammatory adipokines. Anti-inflammatory adipokines including adiponectin, irisin, and ghrelin inhibit the development and progression of NAFLD and NASH, whereas pro-inflammatory adipokines including leptin, resistin, and visfatin promote the development and progression of NAFLD and NASH.

Recently, it has been believed that NAFLD and NASH are caused by the multiple factors [102,103]. Among them, we will focus on adipokines secreted from adipose tissues that provide FA as the major source for NAFLD development [48]. Several adipokines are involved in the pathogenesis and progression of NAFLD [198]. Leptin, resistin, and visfatin play a role in NAFLD development and progression to NASH [199,200,201,202,203,204]. On the other hand, adiponectin, irisin, and ghrelin exert beneficial effects on NAFLD and NASH [205,206,207,208,209,210,211]. Pharmacological agents that affect liver histology and pathophysiology could be influential in theses adipokine levels. It suggests that adipokines can be attractive targets for treatment and can be biomarkers for prediction of NAFLD severity (Figure 5). Adipokines can also play an active role in the development of HCC.

#### 2.3.1. Adiponectin

Adiponectin is an important adipokine that can inhibit NAFLD development. Circulating adiponectin levels were decreased in patients with NAFLD and NASH [220,221,222]. These are inversely correlated with the severity of hepatic steatosis and inflammation. Pioglitazone, an anti-diabetic drug of thiazolidinedione-type, improves liver histology and increases adiponectin levels in patients with NASH [104,125]. However, metformin, the most commonly used anti-diabetic medication, has no significant effects on the liver histology of patients with NAFLD and NASH, and reduces adiponectin levels [107,108,223]. Vitamin E is a potent antioxidant that protects our cells against oxidative stress [224]. It is an alternative medicine that is recommended in NAFLD and NASH. Vitamin E improves liver histology and shows some beneficial effects in non-diabetic patient with NASH, and also seems to increase adiponectin levels [225,226]. However, it has been found to be ineffective alone in NASH patients with type 2 diabetes [226,227]. In mouse models, adiponectin suppresses hepatic lipid accumulation by enhancing FA oxidation and reducing DNL [206,228,229]. It exerts anti-inflammation, anti-fibrotic, and anti-apoptotic effects [229]. Administration of adiponectin improves hepatic steatosis and inflammation [228,229]. Additionally, adiponectin expression is inversely correlated with tumor size and local recurrence [230,231].

#### 2.3.2. Leptin

Leptin is an appetite-suppressing hormone secreted by fat cells. It regulates food intake, body fat, and insulin sensitivity [232]. In animal models, it is thought that it improves lipid metabolism in non-adipose tissues [233]. In the liver, however, it exacerbates hepatic insulin resistance, which results in liver steatosis. It also enhances liver fibrosis [199,233]. Leptin administration can enhance pro-inflammatory and fibrogenic responses in the liver via procollagen I and TGFβ1 [234]. In humans, however, its effects are unclear. Circulating leptin levels are increased in patients with NASH [235,236]. Leptin levels are positively correlated with steatosis severity, whereas it is unclear between leptin levels and the progression of inflammation and fibrosis [235,236,237,238]. Leptin expression is positively correlated with cell proliferation in HCC, as confirmed by proliferation marker protein Ki67 [231].

#### 2.3.3. Resistin

Resistin is a proinflammatory adipocyte-derived mediator of hepatic insulin resistance [239,240]. It is also expressed in liver cells. Resistin is associated with hepatic lipogenesis and liver fibrosis [241]. Circulating resistin levels are increased in patients with NAFLD and NASH, and circulating resistin levels in patients with NAFLD are related to the severity of steatosis, inflammation, and fibrosis [202,242,243]. Increased resistin levels are thought to be associated with insulin resistance. In individuals with NAFLD, pioglitazone treatment improves insulin sensitivity, and decreases plasma resistin levels [244].

#### 2.3.4. Ghrelin

Ghrelin is an anti-inflammatory adipokine. It is an endogenous ligand for growth hormone secretagog receptor with a peptide structure that contains 28 amino acids [245]. In patients with NAFLD, lower ghrelin levels are associated with insulin resistance [246,247]. Plasma ghrelin levels are significantly correlated with liver function. However, ghrelin is not affected by pioglitazone as one of insulin sentizers [45]. During and after NAFLD development, ghrelin administration improves hepatic lipid metabolism, inflammation, oxidative stress, and apoptosis [210]. In mouse models, ghrelin reduces the TG content and the cytokins TNF-α and IL-6, and attenuates lipotoxicity through autophagy sitimulation and NF-kB inhibition [248]. Collectively, ghrelin could be a biomarker for diagnosis and a therapeutic target for treatment of NALFD.

#### 2.3.5. Irisin

Irisin is a myokine secreted from skeletal muscle upon shivering and exercise stimulation [249]. Fibronectin type III domain containing 5 precursors (FNDC5) is the precursor of irisin. FNDC5/irisin promotes the thermogenic program in adipose tisseus through ERK and p38 pathways [250]. It improves glucose homeostasis and insulin resistance, and induces weight loss [251]. Recently, FNDC5/irisin was induced during adipocyte differentiation, and can be over-secreted from human obese visceral (VAT) and subcutaneous (SAT) adipose tissues [252]. It is thought of as a compensatory effect. In line with this, circulating irisin levels are increased in patients with NAFLD, and are positively related with portal inflammation [218]. They are also believed to act as a compensatory effect.

#### 2.3.6. Visfatin

Visfatin is one of the proinflammatory adipokines. Serum visfatin levels are raised in type 2 diabetes and insulin resistant conditions [253,254]. Circulating visfatin levels are also increased in patients with NAFLD, and are associated with the severity of hepatic steatosis and fibrosis [204,255]. However, they are not affected by insulin sensitizers including pioglitazone, rosiglitazone, and metformin [256,257].

## 3. NAFLD/NASH-Derived HCC

### 3.1. The Pathogenesis of NAFLD/NASH-Derived HCC

HCC is the third most common cause of cancer-related mortality [258]. NAFLD and NASH-related HCC is the fastest growing indication for liver transplantation [259,260]. Cirrhosis is only present in approximately 60% of patients with NAFLD and NASH-associated HCC [259]. This suggests that HCC can be induced from NAFLD/NASH without cirrhosis. Therefore, it is thought that “inflammatory factors” will also play a critical role in NAFLD/NASH-derived HCC.

#### 3.1.1. Gut-Derived Endotoxin

As mentioned above, gut-derived endotoxins as alternative inflammatory factors play an important role in the development of NAFLD and NASH. The levels of LPS, known as endotoxins, are also increased in portal and peripheral veins of patients with HCC [261]. They significantly promote the invasive potential and induce the epithelial-mesenchymal transition (EMT), although they also inhibit tumor growth [262]. LPS activates JNK and MAPK via TLR4 in HCC cells, whereas inhibition of JNK/MAPK significantly reduces EMT occurrence [262]. Therefor, the LPS-TLR4 signaling could be one of the promising pathways regulating the progression from NAFLD to NASH to HCC [263].

#### 3.1.2. Adipokines

Adipokines are inflammatory factors related with HCC development. Adiponectin expression in human HCC is inversely correlated with tumor size [230]. It enhances phosphorylation of c-Jun N-terminal kinase (JNK) and activates caspase-3, leading to apoptosis in HCC [230]. Inhibition of JNK phosphorylation prevents anti-apoptotic effects of adiponectin [230]. Adiponectin exerts chmoprotective and hepatoprotective effects via sulfatase2 (SULF2) in HCC [264]. Loss of adiponectin promotes fibrosis and HCC progression in a cholin-deficient NASH mouse model [265]. On the other hand, high levels of circulating adiponectin make it possible to predict the consecutive development of HCC and poor HCC survival [266,267]. Further, adiponectin inhibits the oncogenic effects of leptin on cell proliferation, migration, and invasion in HCC [231].

Leptin expression is increased in both hepatoma tissues and cell lines [268]. Regulatory T-cells (Tregs), effector CD4(+), and CD8(+) T-cells stimulate expression of the leptin receptor (LEPR) in the liver after HCC induction [268]. Macrophage and dendritic cells upregulate LEPR expression on the T-cell. Leptin inhibits Treg activation and function [268]. Increased leptin expression in HCC is associated with the expression of human telomerase reverse transcriptase (hTERT) [269]. Leptin might play a critical role in obesity-related tumorigenesis. Adipokines including adiponectin and leptin represent key players in obesity-related disorders and might be involved in the pathogenesis of NAFLD and HCC.

### 3.2. Diagnostic and Therapeutic Targets in NAFLD/NASH-Derived HCC: Hepatokines

**Figure 6:**
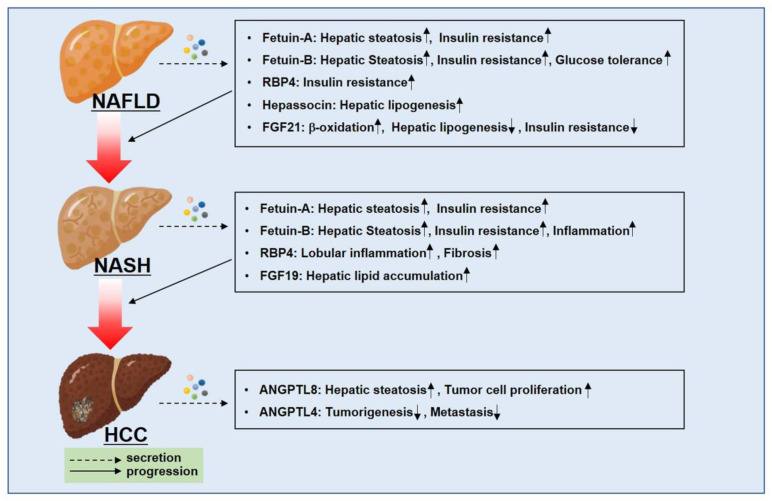
Hepatokines that are secreted from the liver are closely associated with the progression from NAFLD to NASH to HCC. Hepatokines including Fetuin-A, Fetuin-B, RBP4, and FGF19 play an important role in NAFLD and NASH. They are associated with hepatic lipid accumulation, insulin resistance, and inflammatory signaling pathways. Additionally, ANGPTL4 and 8 tend to function in opposite ways in HCC tumorigenesis.

The liver is a secretory organ that releases specific cytokines, termed hepatokines [43]. Adipose tissues in NAFLD, characterized by hepatic TG accumulation, play a critical role in promoting FFA uptake into the liver through lipolysis [48]. Therefore, the role of adipokines from adipose tissues, which provide the major energy source for the development of NAFLD, will be very important in the liver. On the other hand, lipid droplet accumulation itself does not affect inflammation and is considered as simple steatosis. The progression from NAFLD to NASH to HCC needs additional factors such as oxidative stress, mitochondrial dysfunction, and ER-stress [75,270,271]. Another important factor driving NASH in simple steatosis is free non-esterified cholesterol and its oxidized derivatives [272,273,274]. They are cytotoxic and exert synergistic effects with TNF, which is markedly increased in patients with NASH [274]. Therefore, hepatokines secreted from the liver might exert a more potent ability in the progression of NAFLD and NASH to HCC (Figure 6).

#### 3.2.1. α2-HS-Glycoprotein (Fetuin-A and Fetuin-B)

Fetuin-A, one of the liver secreted glycoproteins, is known as the first hepatokine shown to associated with metabolic diseases [275,276]. Fetuin-A is positively associated with hepatic steatosis and insulin resistance [277,278,279]. Its levels are increased in patients with NAFLD, NASH, and type 2 diabetes [280,281]. As an important source of NAFLD development, FFA enhances pro-inflammatory Fetuin-A expression [7]. FFA-induced Fetuin-A functions as an endogenous ligand of Toll-like receptor 4 (TLR4), and exacerbates lipid-mediated insulin resistance [282,283]. FFA can also enhances NF-kB recruitment to the Fetuin-A promoter and increases synthesis and the secretion of Fetuin-A in primary hepatocytes [284]. Pioglitazone significantlly suppresses serum Fetuin-A levels in patients with type 2 diabetes [285]. Pioglitazone inhibits mRNA and protein levels of hepatic Fetuin-A, and oral administration of pioglitazone in mice partially ameliorates insulin resistance with decreases on hepatic fetuin-A expression [286]. These data suggest that Fetuin-A might be a therapeutic target for treatment of NAFLD/NASH and insulin resistance. Additionally, circulating Fetuin-A levels are increased in patients with HCC [287,288]. Fetuin-B might also be an independent indicator of NAFLD development [289]. It induces hepatic steatosis, insulin resistance, and glucose intolerance [277,290]. It decreases AMPK phosphorylation levels and aggravates LXR/SREBP1c-mediate hepatic lipogenesis [291]. On the other hand, circulating Fetuin-A and Fetuin-B levels in patients with NAFLD are negatively associated with liver fibrosis [292,293].

#### 3.2.2. Retinol Binding Protein 4 (RBP4)

The liver plays a central role in vitamin A metabolism. In NAFLD, hepatic vitamin A homeostasis is disrupted [294,295]. RBP4 is a specific retinol/vitamin A carrier protein secreted from the liver. It is also secreted from adipocytes and macrophages [296]. Serum RBP4 levels are associated with NAFLD development [297,298,299]. Circulating RBP4 levels are positively correlated with the body mass index (BMI) and insulin resistance [300,301]. In moderate/severe NASH, high levels of hepatic RBP4 corelated with lobular inflammation and fibrosis scores [302], whereas other studies indicate that both serum and hepatic RBP4 levels negatively correlate with the liver fibrosis stage [299]. In cirrhosis, RBP4 expression improves hepatic glucose production, but not insulin sensitivity [303]. It is true that vitamin A homeostasis is broken and deficient as a result of liver fibrosis and cirrhosis [294]. Importantly, high RBP4 levels could be a marker of NAFLD development, and the lower levels of RBP4 may also be an indicator of the progression ot NASH with fibrosis among NAFLD disease stages [297,298].

#### 3.2.3. Hepassocin (HPS)

HPS, which is known as hepatocyte-derived fibrinogen-related protein 1 (HFREP-1), is a hepatokine that is involved in liver regeneration [304]. In mice and patients with NAFLD, plasma HPS levels are increased [305]. HPS overexpression increased hepatic lipid accumulation, and NAFLD activity scores (NAS), whereas its deletion improves them [305,306]. Serum HPS levels are correlated with the levels of inflammatory cytokines and lipogenic gene expression [306]. HPS-induced hepatic steatosis is triggered through an extracellular signal-regulated kinase 1/2 (ERK1/2)-dependent pathway [306,307,308]. FFA induces HPS expression [309,310]. Oleic acid, the most wildely destributed unsaturated FA, induces HPS expression through signal transducer and activator of transcription 3 (STAT3) signaling [309]. Palmitate, the most abundant saturated FA, induces HPS expression through ER stress-mediated p38 activation by C/EBPβ in primary hepatocytes [310]. Additionally, hepatic HPS expression is increased by partial hepatectomy in mice, and is induced by the hepatocyte nuclear factor 1alpha (HNF1α) through the IL-6/STAT3 pathway [311]. HPS administration protect against liver injury and improves survival in rats with hepatitis [312]. Liver-specific HPS expression is repressed with the downregulation of HNF1α in HCC [311,313].

#### 3.2.4. Fibroblast Growth Factors 19 and 21 (FGF19 and FGF21)

FGF19 and FGF21 are the FGF19 subfamily that requires the Klotho proteins as cofactors. They activate FGFR4 together with Klotho, which is abundantly expressed in hepatocytes [314,315]. FGF19 and FGF21 regulate bile acid, lipid, and the glucose metabolism [314,316].

##### Fibroblast Growth Factors 19 (FGF19)

In NASH, the levels of serum FGF19, fibroblast growth factor receptor 4 (FGFR4), and bile acids are significantly increased, and results in impaired FXR and FGFR4-mediated signaling [317,318]. In patients with NASH, FAF analogue significantly reduces hepatic lipid accumulation. On the other hand, upregulation of FGF19 is associated with the progression, recurrence, and poorer prognosis of HCC [319,320,321]. The β-Klotho proteins are also increased in liver and serum of patients with HCC [321,322].

##### Fibroblast Growth Factors 21 (FGF21)

The hepatokine FGF21 has the beneficial effects on hepatic lipid metabolism. It enhances lipid oxidation, suppresses de novo lipogenesis, and improves insulin resistance by inhibiting mammalian target of rapamycin (mTOR) [323,324,325]. Hepatic FGF21 expression is positively related with adipocity and intrahepatic triglycerids, and its serum levels are significantly increased in patiens with obesity, NAFLD, and type 2 diabetes [326,327,328]. Serum FGF21 levels are increased in obese children with or without NAFLD [329,330]. Elevated serum FGF21 is thought to be able to an independent marker associated with the development of metabolic syndrome [326]. Serum FGF21 levels are increased according to steatosis severity, and are positively correlated with NAFLD activity scores (NASs) [331,332]. Patients with an advanced NASH can be characterized by circulating FGF21 levels combined with inflammatory factors (cytokeratin-18-M30 antigen, IL-1Ra, pigment epithelium-drived factor, and osteoprotegrin) [333]. Increases in serum and hepatic FGF21 levels are observed in cirrhosis and HCC [334].

#### 3.2.5. Angiopoietin-Like Protein 8 (ANGPTL8)

ANGPTL8/betatrophin is circulating a hepatokine that is known as TD26 and lipasin [335]. It is highly expressed in liver and visceral adipose tissue [335,336]. It is associated with hepatic steatosis and increased plasma triacylglycerol levels [336,337,338]. ANGPTL8 overexpression in brown adipose tissue (BAT) enhances lipoprotein lipase activity (LPL) activity and TG uptake [339,340]. Serum ANGPTL8 levels are significantly increased in patients with prediabetes and type 2 diabetes [341]. It has reported that ANGPTL8 is involved in proliferation of pancreatic beta-cells and regulation of glucose and lipid metabolism in mice [342,343,344]. Additionally, ANGPTL8 expression is markedly increased in HCC [337]. It interacts with SREBP-1, and resultingly promotes lipogenesis and tumor cell proliferation in HCC [337,338]. Therefore, it is thought that it is positively correlated with the tumor size. ANGPTL8 requires ANGPTL3 rather than regulating LPL alone [339,345,346]. ANGPTL3 regulates TG metabolism by directly inhibiting LPL [339,347,348]. ANGPTL4, which is abundantly expressed in the liver and adipose tissues, can also regulate TG metabolism by suppressing LPL activity [339,349]. However, ANGPTL4 expression is decreased in HCC, and overexpression of ANGPTL4 suppresses HCC tumorigenesis and metastasis [350].

## 4. Conclusions

In the last 20 years, the proportion of HCC patients with non-viral etiology has been rapidly increasing. For that reason, the importance of NAFLD/NASH-derived HCC has been emerging. Currently, it is true that the treatment of patients with NAFLD/NASH is generally performed using medication for patients with type 2 diabetes and hyperlipidemia. The side effects that appear with long-term use cannot be ignored. Therefore, appropriate therapeutic targets and FDA-approved therapies are urgent. It is thought that the reasons for failing to develop a treatment for patients with NAFLD/NASH despite ongoing attempts are as follows: 1 > unclear pathogenesis, 2 > lack of effects, and 3 > safety problems. Adipose tissue and the liver are the major organs associated with the lipid metabolism. Therefore, it is necessary to observe adipokines and hepatokines, which can be diagnostic and therapeutic targets, along with the signaling pathway targeted by the current treatments. Additionally, it would be good to make an in-depth observation through the classification according to the cause of NAFLD. It will provide an important point of view for controlling the metabolic phenotype from NAFLD to NASH to HCC. Currently, it is acceptable to consider that NAFLD is caused by a concert of various factors including nutritional factors, gut microbiota, and genetic and epigenetic factors, as well as adipokines and hepatokines. In order to find an appropriate treatment, it is necessary to observe various factors in a broader perspective.

## Figures and Tables

**Table 1:** Summary of promising drugs for NAFLD/NASH.

Name of Drug	Mechanism of Action	Metabolic Profile	Liver Histology	Clinical Stage (Title of Trial)	Ref.
Pioglitazone	PPAR γ agonist	Insulin sensitivity(↑) Hepatic TG(↓) ALT(↓) AST(↓) BW(↑)	Steatosis(↓) Ballooning(↓) Inflammation(↓) Fibrosis(↓) NAS(↓)	Phase 4 trial, 2008–2014	[104,105,125,212,213]
Obeticholic acid (OCA)	FXR agonist	Insulin sensitivity(↑) ALT(↓) AST(↓) ALP(↑) HDL-C(↓) LDL-C(↑) BW(↓)	Steatosis(↓) Ballooning(↓) Inflammation(↓) Fibrosis(↓) NAS(↓)	Phase 3 trial, ongoing since 2017 (REGENERATE, REVERSE)	[214,215]
Elafibranor	Dual PPARα/δ agonist	Insulin sensitivity(↑) Plasma TG(↓) ALT(↓) AST(-) ALP(↓) BW(-)	Steatosis(↓) Inflammation(↓) Fibrosis(↓)	Phase 3 trial, ongoing since 2016 (RESOLVE-IT)	[144,150,151]
Aramchol	SCD1 inhibitor	Insulin sensitivity(↑) Hepatic TG(↓) ALT(-) AST(-) BW(-)	Steatosis(↓) Fibrosis(↓)	Phase 3 trial, ongoing since 2019 (ARMOR)	[157]
Liraglutide	GLP-1R agonist	Insulin sensitivity(↑) Hepatic TG(↓) ALT(↓) AST(-) BW(↓)	Steatosis(↓) Ballooning(↓)	Phase 3 trial, ongoing since 2014 (CGH-LiNASH)	[164,216,217]
Selonsertib	ASK1 inhibitor	ALT(↓) AST(↓) BW(-)	Steatosis(↓) Ballooning(↓) Inflammation(↓) Fibrosis(↓) NAS(↓)	Phase 3 trial, ongoing since 2019 (STELLAR3, STELLAR4)	[93]
Simtuzumab (SIM)	LOXL2 monoclonal antibody	ALT(-) AST(-) BW(-)	Fibrosis(-)	Phase 2 trial, 2012–2017	[218,219]
Cenicriviroc (CVC)	Dual CCR2/CCR5 antagonist	ALT(-) AST(-) BW(-)	Inflammation(↓) Fibrosis(↓)	Phase 3 trial, ongoing since 2017 (AURORA)	[196]

((↑): increase, (**↓**): decrease, (-): no significant).
